# Marine Bacteriophages as Next-Generation Therapeutics: Insights into Antimicrobial Potential and Application

**DOI:** 10.3390/v17070971

**Published:** 2025-07-10

**Authors:** Riza Jane S. Banicod, Aqib Javaid, Nazia Tabassum, Du-Min Jo, Md. Imtaiyaz Hassan, Young-Mog Kim, Fazlurrahman Khan

**Affiliations:** 1 Fisheries Postharvest Research and Development Division, National Fisheries Research and Development Institute, Quezon City 1128, Philippines; riza.banicod@nfrdi.da.gov.ph; 2 Interdisciplinary Program of Marine and Fisheries Sciences and Convergent Technology, Pukyong National University, Busan 48513, Republic of Korea; aqibj@pukyong.ac.kr; 3 Marine Integrated Biomedical Technology Center, The National Key Research Institutes in Universities, Pukyong National University, Busan 48513, Republic of Korea; nazia99@pukyong.ac.kr (N.T.); ymkim@pknu.ac.kr (Y.-M.K.); 4 Research Center for Marine Integrated Bionics Technology, Pukyong National University, Busan 48513, Republic of Korea; 5 National Marine Biodiversity of Korea (MABIK), Seochun 33662, Republic of Korea; dmjo@mabik.re.kr; 6 Centre for Interdisciplinary Research in Basic Sciences, Jamia Millia Islamia, New Delhi 110025, India; mihassan@jmi.ac.in; 7 Department of Food Science and Technology, Pukyong National University, Busan 48513, Republic of Korea; 8 Ocean and Fisheries Development International Cooperation Institute, Pukyong National University, Busan 48513, Republic of Korea; 9 International Graduate Program of Fisheries Science, Pukyong National University, Busan 48513, Republic of Korea

**Keywords:** marine bacteriophage, phage therapy, phage–antibiotic synergy, antimicrobial resistance, phage cocktail, phage-derived enzymes

## Abstract

Microbial infections are an escalating global health threat, driven by the alarming rise of antimicrobial resistance (AMR), which has made many conventional antibiotics increasingly ineffective and threatens to reverse decades of medical progress. The rapid emergence and spread of multidrug-resistant bacteria have severely limited treatment options, resulting in increased morbidity, mortality, and healthcare burden worldwide. In response to these challenges, phage therapy is regaining interest as a promising alternative. Bacteriophages, the most abundant biological entities, have remarkable specificity toward their bacterial hosts, enabling them to selectively eliminate pathogenic strains. Phage therapy presents several advantages over conventional antibiotics, which include minimal disruption to the microbiome and a slower rate of resistance development. Among the various sources of phages, the marine environment remains one of the least explored. Given their adaptation to saline conditions, high pressure, and variable nutrient levels, marine bacteriophages mostly exhibit enhanced environmental stability, broader host ranges, and distinct infection mechanisms, thus making them highly promising for therapeutic purposes. This review explores the growing therapeutic potential of marine bacteriophages by examining their ecological diversity, biological characteristics, infection dynamics, and practical applications in microbial disease control. It also deals with emerging strategies such as phage–antibiotic synergy, genetic engineering, and the use of phage-derived enzymes, alongside several challenges that must be addressed to enable clinical translation and regulatory approval. Advancing our understanding and application of marine phages presents a promising path in the global fight against AMR and the development of next-generation antimicrobial therapies.

## 1. Introduction

Microbial infections have emerged as a critical global health challenge, mainly driven by the alarming rise of antimicrobial resistance (AMR). AMR arises when microorganisms, particularly bacteria, develop mechanisms to evade the effects of antibiotics that were once effective. This growing resistance has rendered many conventional treatments increasingly ineffective, threatening to reverse decades of medical progress [1,2,3]. The World Health Organization (WHO) has recognized AMR as one of the top ten global public health threats, cautioning that without coordinated intervention, the world could soon enter a post-antibiotic era in which routine infections and minor injuries may once again become fatal [4,5,6].

The global spread of multidrug-resistant (MDR) pathogens across healthcare systems, communities, and environmental reservoirs, including marine ecosystems, has worsened this crisis [7]. The excessive and improper use of antibiotics, along with the emergence of “superbugs” such as methicillin-resistant *Staphylococcus aureus* (MRSA), vancomycin-resistant *Enterococcus* (VRE), and carbapenem-resistant *Enterobacteriaceae* (CRE), have undermined the efficacy of existing therapies [8,9,10]. Treating MDR infections frequently involves using older, more toxic antibiotics or expensive combination therapies [11]. These limitations underscore the urgent need for novel alternative treatment methods to conventional antibiotics.

One of the most promising alternatives under investigation is bacteriophage therapy [12]. Bacteriophages, or phages, are viruses that specifically infect and lyse bacteria. They are the most abundant biological entities on Earth, outnumbering bacteria by at least 10-fold [13,14,15]. Although discovered in the early 20th century, phage therapy has gained renewed attention in the wake of antibiotic resistance [12,16]. Compared to broad-spectrum antibiotics, phages are highly specific, targeting only particular bacterial strains while preserving the host’s normal microbiota. Their ability to multiply at infection sites, co-evolve with bacterial hosts, and synergize with antibiotics makes them especially promising as therapeutic agents [17,18,19]. As a result, phage applications are being explored not only in clinical medicine but also in agriculture, veterinary science, aquaculture, and food safety [20,21,22].

Among the many sources of phages, the marine environment, which accounts for more than 70% of the Earth’s surface, offers a vast and largely untapped reservoir of phage diversity. These ecosystems host extensive microbial communities, including pathogenic bacteria that are becoming increasingly resistant to antibiotics [23,24,25]. Shaped by distinct saline water conditions, high pressure, and variable nutrient availability, marine bacteriophages commonly exhibit increased stability, broad host specificity, and distinctive lytic mechanisms. These features make them particularly attractive for therapeutic applications in both aquaculture and human health [26,27,28].

Numerous studies have determined the efficacy of marine-derived phages in targeting MDR pathogens, particularly *Vibrio* spp. [29,30,31]. Therapeutic strategies have included the co-administration of phages with antibiotics to hinder resistance development as well as the use of phage cocktails to broaden antibacterial coverage [32]. Advances in synthetic biology have enabled the engineering of phages with improved host specificity, thermal stability, and lytic efficiency [33]. Furthermore, phage-derived enzymes, such as endolysins, are being evaluated for their capacity to degrade bacterial cell walls via mechanisms different from those of traditional antibiotics [34].

Despite their potential, the therapeutic application of marine phages faces a number of challenges. Early phage therapy trials were impeded by issues such as poor standardization, inadequate quality control measures, and inconsistent outcomes, which led to a decline in scientific and clinical interest [12]. Moreover, bacteria are capable of developing resistance to phages, just as they do to antibiotics. Other important considerations include understanding phage pharmacokinetics, optimizing delivery systems, and evaluating interactions with the host immune system to guarantee safe and effective treatment [24,35].

This review provides a comprehensive synthesis of current knowledge regarding the therapeutic potential of marine bacteriophages in combating microbial infections. It examines their diversity, biological characteristics, mechanisms of action, and practical applications in biocontrol and therapy. In addition, the review covers recent innovations in phage engineering and enzyme-based therapies while tackling the challenges that must be overcome for successful clinical and regulatory integration. Broadening our understanding of marine bacteriophages holds transformative potential in the global fight against AMR and in shaping the future of next-generation antimicrobials.

## 2. Diversity and Characteristics of Marine Bacteriophages

Marine phages are the most abundant and genetically diverse biological entities in the ocean, reaching concentrations of up to 10 million viral particles per milliliter of seawater [36]. They play essential ecological roles by shaping the microbial community structure, regulating bacterial population dynamics, influencing biogeochemical cycles, and driving microbial evolution through horizontal gene transfer (HGT) and selective pressure [37].

Both culture-dependent and -independent approaches have revealed extraordinary genomic and morphological diversity among marine phages, including many previously uncharacterized or poorly defined taxa, indicating a vast, largely untapped reservoir of viral biodiversity [38,39]. Phages infecting a wide range of marine bacteria, such as *Roseobacter*, *Flavobacterium*, and *Vibrio*, have been isolated and tested for lytic activity against microbial pathogens [40,41]. These phages display remarkable variability in morphology, host range, genome size, and infection strategy, reflecting adaptation to diverse ecological niches [38]. Metagenomic technologies have further broadened the knowledge of marine viral communities, uncovering novel genomes and infection strategies that were previously inaccessible [39]. This section explores the ecological origins, morphological traits, genomic features, life cycle dynamics, host range specificity, and environmental stability of marine phages, factors critical to understanding their biology and therapeutic potential.

### 2.1. Spatiotemporal Dynamics and Biogeography

Marine phages inhabit a broad range of aquatic environments, from nutrient-rich coastal and estuarine waters to the nutrient-poor open ocean, deep-sea sediments, and subsurface biospheres [37]. Viral abundance generally reflects ecosystem productivity, typically declining from freshwater to marine environments, from coastal to open ocean waters, and from surface to deeper layers [36,37,42]. However, high viral loads can persist in meso- and bathypelagic zones despite severe energy limitations, indicating their critical role in deep-ocean microbial dynamics [43,44].

Deep-sea sediments from Saanich Inlet and the Porcupine Seabight harbor abundant and active viral communities persisting for millions of years, as evidenced by high virus-to-cell ratios in ancient sediment cores [45,46]. The isolation of vB_LagS-V1, a temperate phage infecting *Labrenzia aggregata* at 4000 m in the Mariana Trench, led to the recognition of a new viral family (*Hyphoviridae*) and genus (*Labrenmarinevirus*), highlighting unique evolutionary adaptations, such as auxiliary metabolic genes conferring pressure tolerance, to extreme deep-sea conditions [47]. In the Mediterranean bathypelagic zone, the discovery of 28 viral genomes, some as abundant as surface phages, suggests that deep ocean viruses may be among the most prolific on Earth. Long-term metagenomic studies in the North Pacific Subtropical Gyre have uncovered vast viral diversity and a dominance of lysogenic strategies in mesopelagic zones, reflecting adaptation to low energy and host density [43]. Notably, two novel temperate siphoviruses infecting deep-sea *Roseobacter* clade bacteria, vB_ThpS-P1 and vB_PeaS-P1, represent a newly proposed Mu-like head phage group, pointing to previously unrecognized lineages involved in HGT and diversification within bathypelagic microbial communities [48]. Broader surveys across various bathypelagic ecosystems have also revealed high viral diversity, particularly in chemically active environments like cold seeps and hydrothermal vents, where stable geochemical energy supports highly specialized microbial assemblages [49].

By contrast, the diversity of phages in coastal, estuarine, and surface ocean waters is influenced by seasonal fluxes, salinity gradients, and anthropogenic activities. Cyanophages infecting *Synechococcus* are prevalent in estuaries and adapt to fluctuating environmental conditions via specialized genes [50,51,52]. The first phage infecting the ecologically important *Erythrobacter* genus was isolated from coastal waters off China [53]. Coastal regions worldwide, such as those in South Korea, California, Australia, Greece, and Portugal, harbor phages that can lyse biofilm-forming *Vibrio* species associated with aquaculture disease outbreaks [54,55,56,57,58,59]. In the open ocean, phages targeting cosmopolitan bacterial groups such as SAR116 and T4-like phages make up the dominant components of the viral community [38,60].

Vibrio-specific phages are frequently isolated from aquaculture environments and have demonstrated success in reducing pathogen loads and improving survival in cultured species [22,30,61,62]. Moreover, the widespread detection of phages in seafood samples underscores their prevalence and utility in food safety [21,26,29].

Viral populations display notable temporal fluctuations, often in response to host abundance [44,63]. Interestingly, despite considerable differences in microbial biomass between marine environments, the overall concentration of viruses in the water remains within a relatively narrow range, usually between 10^9^ and 10^11^ particles per liter [64]. The global distribution of viruses remains poorly characterized, with emerging evidence suggesting that, despite some phages being widely distributed, many are habitat-specific or endemic. This spatial heterogeneity is shaped by host availability, environmental factors, and dispersal barriers [44,65,66]. Advancing viral biogeography will require improved taxonomy, standardized detection methods, and broader spatiotemporal sampling.

### 2.2. Morphological Diversity of Phages

**Figure 1:**
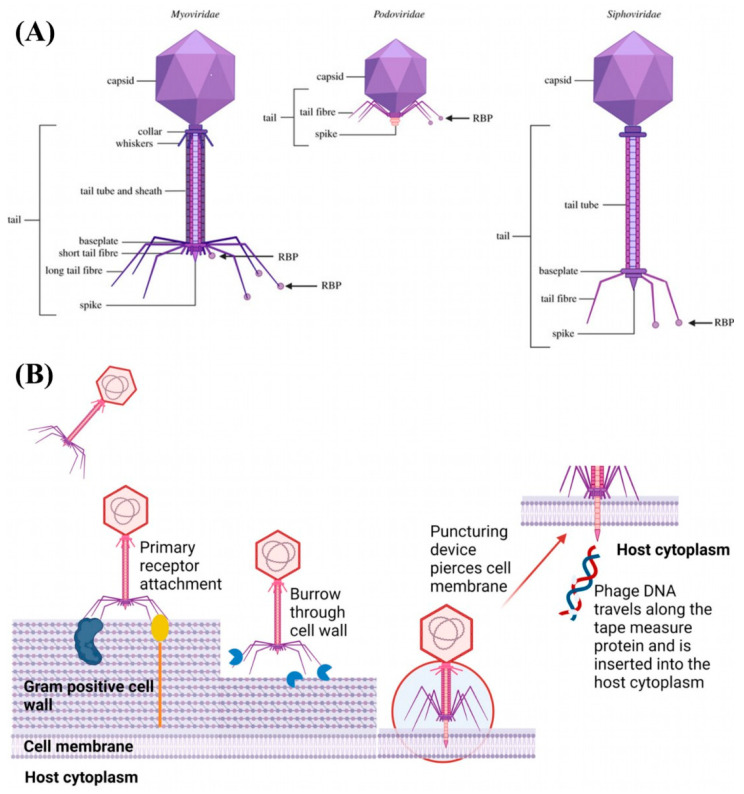
The morphological diversity of tailed bacteriophages and their infection process in bacterial hosts. (**A**) The three principal tailed phage morphotypes, classified by their tail structure: *Myovirus* with a contractile tail and complex baseplate with multiple fibers; *Podovirus*, distinguished by a short, non-contractile tail lacking a baseplate; and *Siphovirus*, which features a long, flexible, non-contractile tail. Receptor-binding proteins (RBPs) are located on tail fibers or spikes and are responsible for recognizing and attaching to specific bacterial surface receptors. Reprinted from [81], Copyright © 2021 by the Authors and the Royal Society under the terms of the Creative Commons Attribution License (http://creativecommons.org/licenses/by/4.0/). (**B**) The generalized infection pathway of a tailed bacteriophage. The process begins with attachment to bacterial surface receptors via tail fibers, followed by penetration of the cell wall and membrane. The phage utilizes a puncturing mechanism to breach the host membrane, after which its DNA is translocated into the host cytoplasm, guided by structural components such as the tape measure protein. Reprinted from [82], Copyright © 2024 by the authors and licensee MDPI, Basel, Switzerland, which is distributed under the terms and conditions of the Creative Commons Attribution (CC BY) license (https://creativecommons.org/licenses/by/4.0/).

Tailed phages, which belong to the class *Caudoviricetes*, represent over 96% of phages described to date. They are distinguished by a protein capsid that encapsulates the viral genome and a tail structure that varies in form and function across three major morphotypes (Figure 1A) [67]. Myoviruses possess icosahedral heads and contractile tails, enabling them to actively inject their genomes into host cells by tail sheath contraction, a process that can penetrate even complex bacterial surface layers [68,69,70]. Siphoviruses possess long, flexible, non-contractile tails with narrow host specificity. Infection and genome delivery are mediated by specialized tail fibers and receptor-binding proteins (RBPs) located at the tail tip, supported by conserved structural modules such as the tape measure protein and tail-associated enzymes [71,72,73,74]. Podoviruses have short, non-contractile tails and utilize compact tail complexes, such as connectors, nozzles, and tail fibers, to breach the host’s outer membrane and facilitate high-pressure DNA ejection [75,76,77]. Infection is initiated by the enzymatic cleavage of polysaccharide receptors, often targeting lipopolysaccharides, with specific tail fiber domains such as the pectate lyase determining host specificity [78,79,80].

Environmental surveys consistently report siphoviruses as the most abundant morphotype in diverse aquatic settings, followed by myoviruses and podoviruses [42], though methodological factors, such as tail fragility and sample handling, can influence the apparent abundance of each morphotype [83].

In addition to tailed phages, marine environments harbor a variety of non-tailed phages with distinct structural forms, including small, polyhedral viruses with single-stranded DNA or RNA genomes and flexible, filamentous particles. They lack a distinct tail structure and are typically encapsulated by a simple protein shell or exhibit elongated, thread-like shapes [84]. Metagenomic studies reveal that single-stranded DNA (ssDNA) phages are genetically diverse and widespread in oceanic environments but remain underexplored, partly due to the limited effectiveness of conventional DNA-binding stains for detecting ssDNA phages [85,86].

### 2.3. Genomic Composition and Functional Traits

Marine phages exhibit remarkable genetic and functional diversity, shaped by ecological adaptation and evolutionary pressures unique to the marine environment. Their genomes, typically composed of either single- or double-stranded DNA or RNA encapsulated within a protein capsid, encode the structural and regulatory elements necessary for infection, replication, and host manipulation [87]. Notably, metagenomic analyses of abundant marine phage genomes reveal substantial reservoirs of uncharacterized genetic material, with up to 70% of predicted coding sequences lacking functional annotation, highlighting the vast, untapped diversity within marine viral communities.

**Figure 2:**
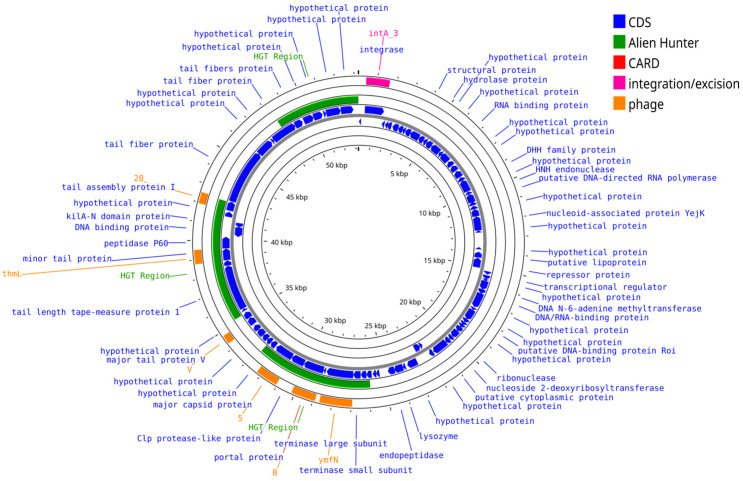
A genetic map of *Vibrio* phage pVa-6 (Genbank accession: KX581097), which was generated using Proksee’s built-in annotation tools and the Genbank annotation file of the phage as input. Phage coding sequences (CDSs) are shown in dark blue. The putative regions exhibiting atypical sequence composition consistent with horizontal acquisition were identified using the AlienHunter tool and are highlighted in green. Functional phage determinants identified through comparison with MobileOG-DB V0.3.0 are categorized and color-coded as follows: canonical phage structural genes are shown in orange, and genes involved in integration and excision are shown in pink. No antibiotic resistance genes (ARGs) were detected upon sequence comparison against the Comprehensive Antibiotic Resistance Database (CARD, V6.0.3), and thus, no red-colored features are shown in the map.

Some of the functional genes identified in marine phage genomes are associated with adhesion, invasion (*csgG* and *fimB*), immune evasion (*clpP*, *clpB*, and *wcbK*), antiphagocytosis (*bplF*, *C*, and *L*), and HGT. Many of these genes are found within genomic islands or adjacent to mobile genetic elements such as transposases and integrases, underscoring the role of phages in gene exchange within marine microbial communities [88,89,90]. The genetic map of *Vibrio* phage pVa-6 (Figure 2) illustrates the modular genome organization, highlighting regions associated with immune invasion (e.g., *Clp* protease like protein), horizontal gene transfer (3 green regions), and integration (*IntA*). No antibiotic-resistant gene was predicted in *Vibrio* phage pVa-6.

**Figure 3:**
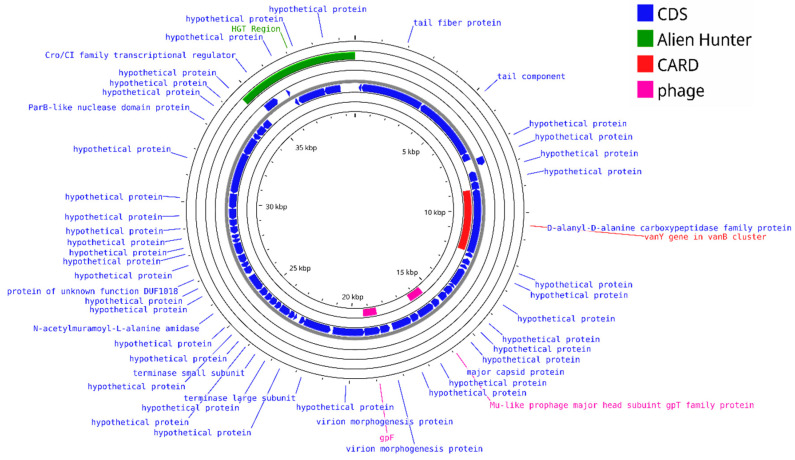
A genetic map of *Thiobacimonas phage vB_ThpS-P1* (Genbank accession: KT381864) generated using Proksee’s built-in annotation tools and the Genbank annotation file of the phage as input. Putative coding sequences (CDSs) are shown in blue. The putative regions exhibiting atypical sequence composition consistent with horizontal acquisition were identified using the AlienHunter tool and are highlighted in green. The antibiotic resistance gene, identified via sequence comparison against the Comprehensive Antibiotic Resistance Database (CARD, V6.0.3), is shown in red. The canonical phage structural proteins identified through comparison with MobileOG-DB V0.3.0 are shown in pink.

Roseophages, which infect *Roseobacter* spp., often possess large genomes (~73–75 kbp) and display unique adaptations, such as a virion-encapsulated RNA polymerase in N4-like podoviruses [41], as well as lysogeny-associated and Mu-like structural modules (e.g., *gpT*, *gp29*, and *gp36*) in deep-sea temperate phages [48]. To further examine these features, a genome map of *Thiobacimonas phage vB_ThpS-P1* (Genbank accession: KT381864) was generated. The map confirms the presence of lysogeny-related genes (*Cro*/*CI* family), a Mu-like prophage major head gene (*gpT* family), and a DUF1018-domain protein (Figure 3).

**Figure 4:**
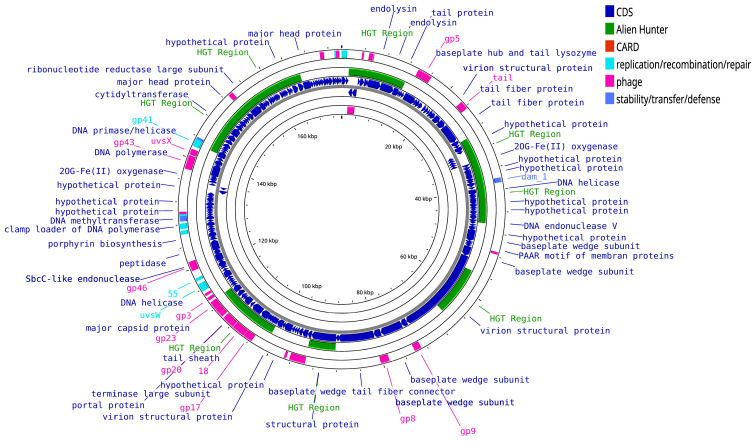
A genetic map of *Cyanophage S-RIM50* isolate RW_29_0704 (Genbank accession: NC_031242), generated using Proksee’s built-in annotation tools and the Genbank annotation file of the phage as input. Phage coding sequences (CDSs) are shown in dark blue. The putative regions exhibiting an atypical sequence composition consistent with horizontal acquisition were identified using the AlienHunter tool and are highlighted in green. Functional determinants identified through comparison with MobileOG-DB are categorized and color-coded as follows: canonical phage structural genes are shown in pink; genes involved in replication, recombination, and repair are shown in cyan; and genes associated with stability, transfer, and defense are shown in blue. No antibiotic resistance genes (ARGs) were detected upon sequence comparison against the Comprehensive Antibiotic Resistance Database (CARD, V6.0.3), and thus, no red-colored features are shown in the map.

One of the most distinctive features of marine phage genomes is the presence of auxiliary metabolic genes (AMGs), which modulate host metabolism during infection. Cyanophages, for example, encode AMGs involved in photosynthesis (*psbA* and *psbD*), electron transport (*PTOX*, *petE*, and *petF*), and stress response, enhancing host function and boosting phage replication, especially in nutrient-limited marine systems. AMGs extend to pathways central to cellular metabolism, including nucleotide biosynthesis (*nrdA*, *nrdB*, *purH*, *purL*, and *thyX*), the pentose phosphate pathway (*ta*lC, *gnd*, and *zwf*), and phosphate acquisition (*phoH*, *phoA*, *pstS*) [91,92,93,94]. The organization and distribution of AMGs in cyanophage S-RIM50 are illustrated in Figure 4.

**Figure 5:**
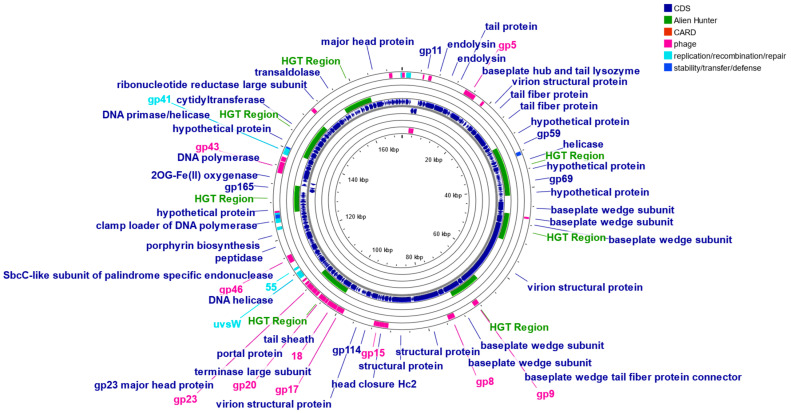
A genetic map of *Synechococcus phage* S-RIM8 A.HR1 (GenBank accession: NC_020486), generated using Proksee’s built-in annotation tools and the GenBank annotation file of the phage as input. Phage coding sequences (CDSs) are shown in dark blue. Putative horizontal gene transfer regions (these regions exhibit atypical sequence composition consistent with horizontal acquisition), identified using AlienHunter, are highlighted in green. Functional determinants identified through comparison with MobileOG-DB are categorized and color-coded as follows: canonical phage structural genes are shown in pink; genes involved in replication, recombination, and repair are shown in cyan; and genes associated with stability, transfer, and defense are shown in blue. No antibiotic resistance genes (ARGs) were detected upon sequence comparison against the Comprehensive Antibiotic Resistance Database (CARD, V6.0.3), and thus, no red-colored features are shown in the map.

Comparative genomic analyses show that T4-like myoviruses infecting *Prochlorococcus* and *Synechococcus* share conserved core genes but exhibit accessory modules that provide adaptability to distinct ecological conditions [95]. The modular genome of a representative *Synechococcus* phage, S-RIM8 A.HR1, demonstrates these features (Figure 5). In contrast, deep-sea siphoviruses display greater genome plasticity, enabling survival under extreme marine conditions [59].

Despite advances in genomics, a substantial fraction of marine viral diversity remains uncharacterized [66]. Metagenomic studies indicate that many marine viral genes lack known homologs, representing a rich resource for discovering novel biological functions and potential biotechnological applications.

### 2.4. Life Cycles

A critical determinant of the therapeutic potential of marine phages is their mode of reproduction, which can follow either a lytic or lysogenic pathway. The lytic cycle dominates in many marine systems, where phages initiate infection by reversibly binding to primary receptors on the bacterial cell surface, followed by irreversible binding to secondary receptors, penetration of the cell envelope, and injection of the viral genome into the host cytoplasm (Figure 1b). This sequence leads to rapid viral replication and culminates in host cell lysis, releasing numerous progeny phages [82,96]. In marine environments, burst sizes can vary, with higher yields being observed under nutrient-rich conditions [97]. The obligate lytic lifestyle is particularly attractive for therapeutic use due to its specificity, efficient amplification at the infection site, and minimal impact on non-target microbiota [82].

In contrast, marine phages such as *Roseophage* R4C and many cyanophages can enter a lysogenic cycle, integrating their genome into the host chromosome or persisting as episomes [98,99]. This dormant state is often favored under environmental stress or low host density, as documented in oligotrophic systems like the Red Sea, where high temperatures and reduced bacterial abundance increase the incidence of lysogeny [100]. Notably, temperate phages display a prolonged latent period and larger burst size compared to strictly lytic counterparts, suggesting an adaptive advantage under resource-limited conditions [101]. Shitrit et al. [102] revealed that some T7-like cyanophages are capable of transient genome integration in cyanobacteria, such as *Synechococcus* and *Prochlorococcus*, but do not establish stable lysogeny, indicating that not all integration events in marine cyanophages result in classical lysogenic cycles. Certain marine phages are also capable of establishing chronic infections, as observed with phages infecting *Cellulophaga* spp., whereby new virions are continuously released from the host without inducing cell lysis [103].

Environmental cues, such as nutrient availability, salinity, aeration, ultraviolet radiation, temperature, pollutants, and host density, play decisive roles in determining life cycle transitions [104,105,106,107]. For instance, high salinity can drive marine phage ΦHSIC from lysogeny to lysis, with phage numbers increasing as NaCl and MgSO_4_ concentrations rise [105]. Field studies in Senegal have similarly shown that increased salinity correlates with higher phage abundance but reduced diversity, reflecting selective pressures in marine systems [108]. Moreover, some phages, like *Vibrio* phage 882, have evolved to sense host quorum signals, allowing them to fine-tune their lytic–lysogenic decisions in response to host population dynamics [109].

**Figure 6:**
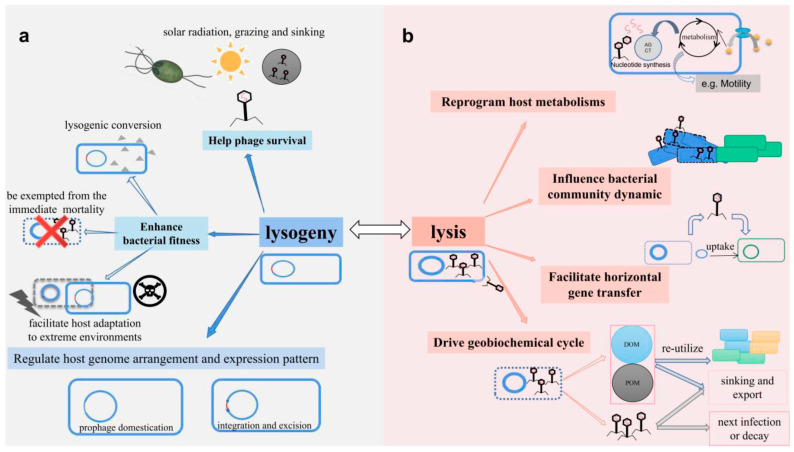
Ecological impacts of lysogenic–lytic transitions in marine phages. (**a**) The shift from the lytic to lysogenic cycle shields free phages from environmental hazards such as solar radiation, grazing by nanoflagellates, and removal via aggregation and sinking. Lysogeny increases host bacterial survival by providing immunity from immediate lysis and supports adaptation to extreme or fluctuating marine conditions. Moreover, lysogeny influences the host genome by integrating prophage DNA, with potential for excision and rearrangement. (**b**) Transitioning from lysogeny to lysis enables prophages to redirect host metabolism toward phage production, impacts microbial community composition through host cell lysis, promotes horizontal gene transfer, and fuels oceanic biogeochemical cycling via the release of cellular contents. Reprinted from [101], Copyright © 2022 by the authors and licensee MDPI, Basel, Switzerland, which is distributed under the terms and conditions of the Creative Commons Attribution (CC BY) license (https://creativecommons.org/licenses/by/4.0/).

Marine phages also face unique ecological challenges, such as solar radiation, which accelerates the decay of free phage particles in surface waters, prompting a higher frequency of lysogeny in offshore, well-lit waters, as observed in the Gulf of Mexico [110]. They are also vulnerable to predation by nanoflagellates and removal via attachment to sinking aggregates. Lysogeny, as depicted in Figure 6a, offers a refuge from these environmental threats, enhances host fitness, and facilitates adaptation to extreme marine conditions [23,101]. Physical barriers, such as fluctuating salinity and variable host receptor availability, can further limit successful lytic infection and favor lysogenic persistence [101].

When environmental conditions improve or specific triggers arise, prophages can be induced to enter the lytic cycle, reprogramming host metabolism to favor phage replication, thus influencing the microbial community structure, promoting HGT, and driving the cycling of organic matter and nutrients, a process central to “viral shunt” (Figure 6b) [96,111]. Furthermore, lysogenic phages can act as genetic reservoirs, transferring AMG to hosts that would enhance their ecological fitness in nutrient-limited marine environments [112].

### 2.5. Host Range and Specificity

The phage host range refers to the specific bacterial strains, species, or genera it can infect and replicate within, a factor fundamental to assessing both ecological roles and therapeutic potential, particularly in marine environments characterized by high microbial diversity, antibiotic resistance, and polymicrobial infections [113]. The definition of “broad host range” varies as it may refer to infectivity across multiple strains of a single species [57,61] or across different species or genera [59,114]. More recently, distinctions have been made between phenotypic broad host range (individual phages infecting multiple hosts) and genotypic broad host range (infectivity distributed across a genetically diverse population) [115].

Host range is primarily determined using culture-based methods such as spot tests and plaque assays, with the latter providing more precise validation of productive infection [113,116,117]. Advances in sequencing and computational methods are improving host range assessment but are still constrained by host cultivability and methodological sensitivity [115]. Isolation strategy also impacts the observed host range as single-host enrichment typically yields narrow-range phages, while the use of multiple hosts can recover phages with broader activity [118].

From an evolutionary perspective, host range is shaped by mutations and recombination in RBP and other adaptive mechanisms, though a broad host range may experience trade-off with reduced infectivity or stability on individual hosts [115,119,120,121]. In oligotrophic marine environments, a broader host range can be advantageous by increasing the likelihood of encountering susceptible bacteria [122]. For therapeutic purposes, the host range informs phage selection. Narrow-range phages offer high specificity but may be insufficient in polymicrobial infections [123], whereas broad-range phages or cocktails provide wider coverage and reduce the risk of resistance development [124]. This is particularly important in aquaculture systems, where rapid and broad-spectrum bacterial control is often needed [125].

Empirical studies illustrate the variability of host range among marine phages and its relevance for therapy. For instance, phage vB_VpaP_MGD2 lysed approximately 90% of antibiotic-resistant *Vibrio parahaemolyticus* strains, demonstrating broad host infectivity and significant potential for aquaculture applications [126]. Conversely, vB_VnaS-L3 showed a narrow host range, infecting only about 7% of tested *Vibrio natriegens* strains [127]. Similar patterns are observed with *Vibrio harveyi* phages from shrimp aquaculture, where most infect over two-thirds of host strains, while some, like VHM1, are limited to a third [128]. These findings underscore the need for thorough host range characterization to ensure therapeutic efficacy and minimize unintended impacts in phage-based interventions.

### 2.6. Environmental Stability and Adaptation

Environmental stability is a key factor determining the survival, infectivity, and practical application of phages in marine and aquaculture systems. Temperature, pH, and salinity significantly influence phage performance by affecting host–phage interactions and persistence in diverse environments [126]. Many marine phages demonstrate remarkable tolerance to environmental stressors, which enhance their suitability as biocontrol agents [26,29,59,127]. Most therapeutically relevant marine phages exhibit optimal stability between 30 °C and 50 °C [126,129,130], with some, such as phage SIO-2, remaining viable at temperatures of up to 65 °C [59]. Similarly, several phages retain high infectivity within pH ranges of 3.0 to 10.0, although activity generally declines outside this range [59,126,131]. Salinity is also crucial as many marine phages require specific ionic conditions for stability and infectivity [132,133]. Advances such as polymer-based stabilization are being explored to improve phage resilience under fluctuating or harsh conditions [134]. As environmental factors such as temperature gradients and salinity shifts shape phage–host dynamics, thereby modulating infection efficiency, bacterial resistance development, and lysogeny rates [135], understanding and engineering phages for environmental resilience is essential for successful therapeutic deployment.

## 3. Marine Bacteriophages as a Therapeutic Alternative

The widespread emergence of AMR has greatly compromised conventional antibiotics, prompting a global call for alternative antimicrobial strategies [1]. Bacteriophages, or viruses that specifically infect and destroy bacterial cells, have emerged as one of the most promising candidates. Unlike antibiotics, phages exhibit a high degree of specificity, thus selectively eliminating pathogenic strains without significantly affecting commensal microbiota or host cells [12]. Obligate lytic phages, in particular, are ideally suited for therapeutic use as their infection cycle culminates in the lysis of the bacterial host and the release of new phage particles, thus amplifying therapeutic efficacy at infection sites [136].

In general, phage therapy offers several advantages over traditional antibiotics, including their self-replication and auto-dosing capabilities in the presence of target pathogens, natural clearance upon the elimination of bacterial populations, minimal off-target effects due to their narrow host specificity, and adaptability to evolving bacterial resistance mechanisms [137]. They are also rapid and cost-effective to isolate, with resistance development in target bacteria occurring at a rate approximately ten times slower than observed with antibiotics. Since phages do not infect eukaryotic cells, they are considered safe, with studies consistently reporting only minor or negligible side effects [138].

Marine ecosystems exert strong selective pressures, driving phage adaptation for survival in conditions characterized by high salinity, variable temperature, and diverse nutrient availability [96,139,140]. Consequently, marine phages typically display enhanced genetic diversity, greater environmental stability, and robust mechanisms for biofilm disruption [141,142]. These characteristics make them particularly effective against prominent marine pathogens, such as *Vibrio* spp., which frequently cause significant disease outbreaks in aquaculture [143,144]. While this section predominantly focuses on marine phages, selected terrestrial or freshwater studies are included for comparative insights or to underline overarching therapeutic principles.

### 3.1. Phage Formulations

Phage formulation and delivery strategies influence treatment success and resistance management [145]. Monophage formulations employ a single phage isolate and often target specific pathogenic bacterial strains. For instance, Li et al. [127] demonstrated that marine phage vB_VnaS-L3 selectively infected *V. natriegens* strains with an exceptionally large burst size (~890 PFU/cell), significantly reducing mortality in juvenile abalones compared to traditional antibiotic treatments with oxytetracycline. Similarly, monophages pVa-21 effectively controlled *V. alginolyticus*, showing potent anti-planktonic and anti-biofilm activities [54], whereas monophage ϕVP-1 specifically disrupted biofilm formation with pathogenic strains of *V. parahaemolyticus* [27]. Challenging the assumption that monophages are narrowly specific, Baudoux et al. [59] reported that *Vibrio* phage SIO-2 can lyse multiple strains within the *Harveyi* clade. Similar broader host range and stability over a wide range of temperature and pH values have been demonstrated by other monophages such as vB_VpaP_MGD2 isolated from clam [126] and vB_VpaS_OMN from the Atlantic Sea [130].

While monophage therapy exerts minimal effects on beneficial flora due to its high specificity, this same characteristic can hinder its effectiveness against diverse or rapidly evolving bacterial populations [146]. The narrow host range not only limits the coverage of targeted pathogens but also increases the risk of rapid bacterial resistance [147]. In light of these limitations, phage cocktails, comprising mixtures of multiple phages targeting a broader array of bacterial strains, have emerged as the standard therapeutic approach, particularly for managing polymicrobial infections that are prevalent in aquaculture systems [125]. Tao et al. [29] demonstrated that a cocktail consisting of vB_VpaS_1601 and vB_VpaP_1701 obtained from oysters completely inhibited *V. parahaemolyticus* growth within 12 h and reduced biofilm formation by up to 78%, outperforming individual phages, which inhibited biofilms by roughly 54% and 65%, respectively. Further studies have shown that phage cocktails effectively reduce *Vibrio* biofilms and planktonic cells in various aquatic species, including shrimp [144], sea cucumbers [148], and fish [57]. These formulations have proven successful in inhibiting pathogenic *Vibrio* growth, improving the survival rates of aquatic animals, and maintaining product quality [149]. Many phage cocktails exhibit broad host ranges, high lytic efficiency, and notable biofilm dispersal capabilities while also reducing the emergence of phage-resistant bacterial strains [125,150]. However, it is important to note that some cocktails have demonstrated the capacity for generalized transduction, potentially facilitating the horizontal transfer of antibiotic resistance or virulence factors among *Vibrio* populations [61].

Recent evidence suggests that phage cocktails may not fully curb resistance as bacterial populations can evolve resistance to all cocktail components within a timeframe similar to monophage exposure. Mechanisms include mutations in phage receptor genes [151], the acquisition of mobile phage defense elements [152], and metabolic adaptations [153]. While phage cocktails can initially prevent *Vibrio* infections, bacteria rapidly develop resistance, often within days [154]. As an alternative approach, Li et al. [154] proposed a novel method wherein phage-resistant bacterial mutants are used as new hosts to isolate additional phages with distinct infection strategies, enabling the rapid assembly of more robust and complementary phage cocktails. Sequential phage therapy has also shown potential for more effectively delaying resistance emergence. This technique, demonstrated primarily against *Pseudomonas* infections, involves administering distinct phages in a staggered sequence, typically at 24 h intervals, thereby applying temporal selection pressures that hinder the simultaneous emergence of cross-resistance. It may reduce abrupt endotoxin release from massive bacterial lysis, offering improved safety profiles in clinical contexts [155,156]. While this technique has been demonstrated primarily in *Pseudomonas*, its principles can be adapted for *Vibrio* management in aquaculture.

### 3.2. Mechanisms of Action of Lytic Marine Phages

Lytic marine phages employ diverse and highly specialized molecular strategies to infect and rapidly lyse their bacterial hosts, particularly *Vibrio* species, which are prevalent in marine environments. These phages demonstrate remarkable efficiency in host recognition, genome injection, intracellular replication, and cell lysis, contributing to their potential as precise and environmentally safe biocontrol agents. The infection process begins with the recognition and adsorption of the phage to the host cell surface. This step is facilitated by RBPs located on phage tail fibers or spikes, which enable specific interactions with bacterial surface structures such as lipopolysaccharides, flagella, and outer membrane proteins [82]. Phage SSP002, for instance, utilizes the flagella of *V. vulnificus* as its primary receptor. The specificity of this interaction was confirmed by studies showing that deletion mutants lacking flagellar genes became resistant to infection, while complementation restored susceptibility. The three ORFs located between the tail tape measure and tail assembly protein genes in SSP002 are believed to contribute to its host specificity at the species level [55].

Following irreversible adsorption, genome injection mechanisms vary depending on phage morphology. In myovirus phages such as *Vibrio* phage XM1, the contractile tail sheath drives the tail tube through the outer and inner membranes of *Vibrio* cells. This penetration is assisted by a specialized cell-piercing protein (gp14), which is homologous to the phage SN cell-piercing protein known for enzymatically degrading the peptidoglycan layer [157]. Similarly, ϕVP-1, which infects *V. parahaemolyticus*, encodes a tail-associated lysozyme (ORF183) with *N*-acetylmuramidase activity that not only facilitates genome delivery but also contributes to the degradation of the host’s biofilm matrix [27]. Siphoviruses, with their long, flexible, non-contractile tails, typically initiate infection through tail fiber-mediated recognition of specific host receptors, followed by genome injection aided by enzymatic tail tip domains that degrade surface barriers. This is exemplified by the marine siphovirus TW1, whose six trimeric tail spikes (gp19) specifically bind to and enzymatically degrade host surface polysaccharides, while a tail-associated lysozyme (gp27) likely facilitates penetration of the peptidoglycan layer, collectively enabling efficient DNA delivery into the bacterial cell [158]. Podoviruses, characterized by their short, non-contractile tails, initiate genome injection upon tail fiber binding to the host cell surface, as shown in phage P-SSP7 infecting *Prochlorococcus marinus*, where this interaction triggers conformational changes at the portal vertex complex, including opening of the nozzle valve (gp12), thereby allowing DNA delivery through the short tail channel into the host cytoplasm [159].

Once inside the host, marine phages initiate rapid replication characterized by short latent periods and high burst sizes. For example, ϕVP-1 exhibits a remarkably short latency and a large burst size, enabling swift host cell lysis and dissemination [27]. Similarly, vB_VpaP_MGD2 completes its lytic cycle in approximately 10 min and releases around 244 plaque-forming units (PFUs) per infected cell. Other marine phages, such as vB_VpaS_1601 and vB_VpaP_1701, demonstrate similar kinetics, adsorbing rapidly within 30 min and achieving high burst sizes, which makes them suitable for prompt population control of *Vibrio* spp. [29].

The culmination of the infection cycle is mediated by a dedicated lysis module, typically composed of holins and endolysins. Holins are responsible for the timed permeabilization of the cytoplasmic membrane, while endolysins enzymatically degrade the peptidoglycan cell wall from within. For instance, phage vB_VpaP_MGD2 encodes a holin (ORF_11) and an endolysin (ORF_3) that work in tandem to disrupt the bacterial cell envelope [126]. Similarly, vB_VpaS_1601 and vB_VpaP_1701 harbor multi-gene lysis modules (orf23, orf30, orf78, and orf2) that coordinate the enzymatic breakdown of the host cell wall [29]. CAU_VPP01 also contains a functionally annotated lysis module among its 114 predicted ORFs, supporting efficient host cell lysis [149].

Phage activity also extends to the disruption of bacterial biofilms, which are notoriously difficult to eradicate due to their dense extracellular matrix and altered gene expression profiles. The marine phage CAU_VPP01, targeting *V. parahaemolyticus*, has been shown to suppress the expression of *flaA* (flagellar gene), *vp0962* (biofilm-associated protein), and *luxS* (quorum sensing regulator), thereby weakening the structural and regulatory integrity of the biofilm. Confocal microscopy and COMSTAT analyses confirmed significant reductions in biomass, thickness, and roughness following phage treatment [149]. Likewise, ϕVP-1 demonstrates potent biofilm-disrupting properties, attributed to enzymes within its tail that degrade extracellular polysaccharides [27].

Marine phages exhibit a range of structural, genomic, and ecological adaptations that enhance their infectivity, stability, and suitability for biocontrol applications. Some possess expanded genomic regions with hyperplastic inserts that are believed to improve phage fitness and confer morphological diversity [59], while others demonstrate remarkable stability under diverse environmental conditions, including moderate UV exposure, as well as salinities and temperatures of up to 40 ppt and 40 °C, respectively [160]. Despite its moderate burst size (~6.76 PFU/infected cell), phage BPVP-3325 exhibits a broad host range and environmental resilience across pH 5–10 and temperatures ranging from 10 to 40 °C, supporting its potential application in aquaculture and food safety [161]. Similarly, the VV-series phages (VV1–VV4) exhibit a stepwise intracellular replication process, with a relatively long latent period (2–3 h), followed by a rapid rise phase and eventual host cell lysis, an infection profile that may enable controlled, sustained phage activity in complex microbial environments [31]. Genomic analyses across these therapeutic phages consistently reveal the absence of undesirable elements such as lysogeny, virulence, and antibiotic resistance genes, highlighting their safety for biocontrol and therapeutic use.

### 3.3. Application of Marine Phage Therapy

Phage therapy is once again drawing attention as a promising solution to the escalating crisis of bacterial disease and antimicrobial resistance, especially in aquaculture where bacterial pathogens are a leading cause of disease outbreaks and economic losses [12]. A growing body of evidence highlights the unique efficacy and versatility of marine phages in the therapeutic management of marine aquaculture diseases.

Among the most well-documented targets are *Vibrio* species, which are prominent marine pathogens. Lomelí-Ortega et al. [160] isolated the *Vibrio* phage vB_Vc_SrVc9 from the hepatopancreas of Pacific white shrimp (*Penaeus vannamei*) affected by acute hepatopancreatic necrosis disease (AHPND). This phage substantially reduced *V. campbellii* loads and increased the survival of experimentally challenged brine shrimp (*Artemia franciscana*) without disrupting beneficial microbiota, demonstrating both high specificity and ecological safety. Similarly, phages isolated from aquaculture farms and seafood samples have shown effective control of *V. alginolyticus* in brine shrimp, which are commonly used as live feed, and in shrimp and oyster culture systems, achieving significant pathogen reduction while preserving the host’s natural microbiome [58,62,131]. Zhu et al. [147] reported that phage XC31 effectively treated yellow spot disease in commercially cultivated seaweed (*Pyropia haitanensis*) by eliminating the causative *Vibrio mediterranei*. Remarkably, the phage not only cleared the infection but also restored photosynthetic efficiency and antioxidant function in the seaweed host.

Marine phage therapy has also demonstrated potential against a wider range of pathogens in various host systems. For instance, the lytic phage BONAISHI, isolated from coral reef waters in Van Phong Bay, Vietnam, effectively infected multiple strains of *V. coralliilyticus* and mitigated damage to *Symbiodinium* cells, indicating its application for managing coral diseases [162]. In bivalve depuration, phages phT4A and ECA2, when applied in static and recirculated seawater systems, significantly reduced *Escherichia coli* concentrations in both artificially and naturally contaminated cockles, thereby shortening depuration time and improving food safety [163]. Similarly, phages phSE-2 and phSE-5 were shown to decrease *Salmonella enterica serovar Typhimurium* by up to 2.0 log CFU/g in *Cerastoderma edule*, highlighting phage-based strategies as viable alternatives to traditional depuration methods [164]. The anti-*Lactococcus garvieae* phage PLgY-16 demonstrated high in vivo persistence in yellowtail (*Seriola quinqueradiata*) and significantly improved fish survival following bacterial challenge [165]. Phages isolated from fish culture systems also reduced mortality in Japanese flounder (*Paralichthys olivaceus*) experimentally infected with *Streptococcus iniae*, with therapeutic effects evident even when administered 24 h after infection [166]. In addition, the marine phage Str-PAP-1, isolated from olive flounder, showed strong lytic activity against *Streptococcus parauberis*. When delivered via feed, it improved fish growth and survival while reducing pathogen load, underscoring its utility as an environmentally friendly antibiotic alternative [167]. Other marine phages with demonstrated or promising therapeutic potential are summarized in Table 1.

In contrast, clinical phage therapy has predominantly relied on phages isolated from terrestrial or human-associated environments, such as hospital sewage and wastewater treatment plants. These phages are typically targeted against MDR strains of *E. coli*, *Pseudomonas aeruginosa*, and *Staphylococcus aureus* [168,169,170]. While their relevance is critical in human medicine, the environmental origin of these phages often reflects the niche-specific co-evolution of phage–host interactions.

Nevertheless, the methodologies and strategies developed in marine phage therapy have begun to inform clinical research, particularly regarding phage selection, delivery routes, and host specificity. The overarching principle remains consistent, with phage therapeutic potential being deeply embedded in their co-evolutionary relationships with bacterial hosts. This principle holds true across environments; wherever pathogenic bacteria exist, phages capable of specifically infecting and lysing these bacteria are highly likely to be found [12].

Given the urgent global need to address antibiotic resistance, leveraging the natural diversity and specificity of phages across ecosystems is imperative. Expanding the integration of marine phage therapy into both clinical and aquaculture disease management could drastically reduce reliance on antibiotics, offering an environmentally sustainable, economically viable, and highly effective approach to pathogen control.

**Table 1:** Overview of marine bacteriophages and their therapeutic application against microbial infections.

Phage Name	Source	Target Pathogen	Phage Morphotype	Genome Type	Genome Size (kb)	Accession No.	Host Range	Stability	Application Area	Reference
PLgY-16	Diseased yellowtail (*Seriola quinqueradiata*)	*Lactococcus garvieae*	Siphoviridae	dsDNA	NA	NA	Broad	pH > 3.5	Yellowtail aquaculture	[165]
PETp9 and PVHp5	Dead turbot (*Scophthalmus maximus*)	*Edwardsiella tarda*, *Vibrio harveyi*	-	-	-	-	Broad	-	Prevention of ascites and bacterial infection in turbot aquaculture	[171]
Str-PAP-1	Olive flounder	*Streptococcus parauberis*	Siphoviridae	dsDNA	36.6	NA	Broad	Stable as a dietary supplement	Prevention and treatment of *S. parauberis streptococcosis* in olive flounder via dietary supplementation	[167]
*S. iniae* phage isolates	Fish culture environment	*Streptococcus iniae*	-	-	NA	NA	Broad	-	Phage therapy for *streptococcosis* in fish	[166]
phT4A	Bivalve culture	*E. coli*	-	-	NA	NA	Narrow	-	Bivalve depuration (cockles)	[163]
BONAISHI	Coral reef water (Van Phong Bay, Vietnam)	*Vibrio coralliilyticus*	Myoviridae	dsDNA	303	MH595538	Narrow	pH 3–10; 4–50 °C;	Coral disease biocontrol/phage therapy	[162]
Vp1 Vp3 Vp5 Vp7 Vp9	Shrimp pond water and sediment	*Vibrio parahaemolyticus*	Myovirus	dsDNA	NA NA NA NA NA	NA NA NA NA NA	Narrow	-	Shrimp aquaculture	[172]
uVh1 uVh2 uVh3 uVh4	Shrimp hatcheries	*V. harveyi*	Siphovirus Siphovirus Podovirus Siphovirus	dsDNA	85 * 58 * 64 * 107 *	NA NA NA NA	Broad	-	Shrimp hatcheries	[61]
SIO-2	Coastal Pacific surface waters	*Vibrio* sp. SWAT3, *V. harveyi* ATCC BAA-1116, and *Vibrio campbellii* ATCC 25920	Siphovirus	dsDNA	80.6	PRJNA42177	Broad	−196–65 °C; pH 3–10	Biocontrol agent	[59]
P3K P4A P7A P8D P9C	Abalone farm	*Vibrio owensii*	Siphovirus	dsDNA	31 * 48 * 41 * 30 * 31 *	NA NA NA NA NA	Narrow	≤50 °C; pH 5–9	Aquaculture industry	[173]
SSP002	Seawater sample from Yellow Sea	*Vibrio vulnificus*	Siphovirus	dsDNA	80.8	JQ801351	Narrow	20–50 °C; pH 4–11	Food industry	[55]
VP-1 VP-2 VP-3	Semi-intensive aquaculture system	*V. parahaemolyticus*	Podovirus	dsDNA	NA	NA	Narrow	-	Aquaculture industry	[150]
φSt2 φGrn1	Water samples from north coastline of Crete, Greece	*Vibrio alginolyticus*	Myovirus	dsDNA	250.5 248.6	KT919973 KT919972	Broad	-	Aquaculture (hatcheries)	[57]
P4A P4F	Abalone farm	*Vibrio* spp.	Siphovirus	dsDNA	49 * 44 *	NA NA	Narrow	-	Marine aquaculture	[30]
VhKM4	Marine aquaculture	*V. harveyi* VHJR7	Myovirus	dsDNA	NA	NA	Broad	-	Aquaculture industry	[22]
VHM1 VHM2 VHS1	Water and sediment samples from shrimp ponds and coastal areas in southeast coast of India	*V. harveyi*	Myovirus Myovirus Siphovirus	dsDNA	55 * 66 * 81.5	NA NA JF713456	Broad	4–50 °C; pH 4–10	Shrimp aquaculture	[128]
PhVh6	Shrimp pond water	*V. harveyi*	Siphovirus	dsDNA	NA	NA	Broad	25 to 65 °C; pH 2–14; 15 to 45 ppt	Biocontrol agent in shrimp aquaculture	[174]
VV1 VV2 VV3 VV4	Hatchery tank water, shrimp culture pond water, and WSSV uninfected *Penaeus monodon*	*V. vulnificus*	Tectivirus	dsDNA	NA NA NA NA	NA NA NA NA	Broad	≤60 °C; pH 6–11	Biocontrol agents against *Vibriosis* in shrimp aquaculture environment	[31]
vB_VhaS-a vB_VhaS-tm	Water and oyster tissue sample	*V. harveyi*	Siphovirus	dsDNA	82 59	KX198614 KX198615	Narrow	≥23 °C	Abalone aquaculture	[175]
Vpms1 A3S Aie F8 F12	Shrimp aquaculture	*V. parahaemolyticus* and *V. harveyi*	Podovirus Siphovirus Levivirus Levivirus Podovius	dsDNA dsDNA ssDNA ssDNA dsDNA	42.3	NC_021776 NA NA NA NA	Narrow	28–30 °C	Brine shrimp (*Artemia franciscana*) production	[125]
vB_VpaS_OMN	Atlantic sea	*V. parahaemolyticus*	Podovirus	dsDNA	42.2	NC_048167	Broad	≤50 °C; pH 5–9	Oyster decontamination	[130]
pVa-21	Seawater samples from West Sea of South Korea	*V. alginolyticus*	Myovirus	dsDNA	232.0	KY499642	Narrow	4–35 °C; pH 7	Biocontrol agent	[54]
VP06	Seawater, sediment, and animals (oysters and clams)	*V. parahaemolyticus*	Siphovirus	dsDNA	75.9	MG893203	Broad	4–37 °C; pH 7–11	Aquaculture systems	[176]
vB_VpaP_MGD2	Clam (*Meretrix meretrix*)	*V. parahaemolyticus*	Podovirus	dsDNA	45.1	MK820013	Broad	30–50 °C; pH 3–10	Shrimp production	[126]
Φ-5 Φ-6 Φ-7	Oyster hatchery	*V. alginolyticus*	Myovirus	dsDNA	238.1 NA NA	MK358448 NA NA	Broad	-	Biocontrol agent in oyster hatcheries	[62]
ϕVP-1	Shrimp pond water	*V. parahaemolyticus*	Myovirus	dsDNA	150.8	MH363700	Narrow	≤50 °C; pH 5–9	Biocontrol agent of biofilm-forming strains	[27]
pVco-5 pVco-7 pVco-14	Oyster hatchery	*Vibrio coralliilyticus*	Podovirus	dsDNA	74.3 75 59.4	NC_055717 PP107878 MW114771	Narrow	4–37 °C; pH 7–9	Biocontrol agent in marine bivalve hatcheries	[146,177]
Φ-1 Φ-2 Φ-3 Φ-4	Marine water samples from Sunshine Coast region of Queensland, Australia	*Vibrio* spp.	Myovirus	dsDNA	NA 242.4 NA NA	NA MK368614 NA NA	Broad	-	Microalgae feed for oyster hatcheries	[56]
vB_VpS_BA3 vB_VpS_CA8	Sewage from aquatic product market	*V. parahaemolyticus*	Siphovirus	dsDNA	58.6 58.5	MN175679 MN102376	Narrow Broad	20–40 °C; pH 5–7	Biocontrol method for multidrug-resistant *V. parahaemolyticus*	[178]
vB_Vc_SrVc9	Hepatopancreas of Pacific white shrimp (*Penaeus vannamei*)	*V. campbellii*	Podovirus	dsDNA	43.2	LR794124	Broad	20–40 °C; 10 ppt; Sensitive to UV	Brine shrimp (*A. franciscana*) aquaculture	[160]
Phage XC31	Marine environment	*Vibrio mediterranei* 117-T6	-	dsDNA	290.5	MK308674	Narrow	-	Seaweed culture as biological control strategy for yellow spot disease	[147]
Vp33 Vp22 Vp21 Vp02 Vp08 Vp11	Fresh seafood	*V. parahaemolyticus*	Podovirus Podovirus Podovirus Podovirus Siphovirus Siphovirus	dsDNA	NA NA NA NA NA NA	NA NA NA NA NA NA	Narrow	−20–50 °C; pH 5–11	Food safety	[21]
OY1	Sewage from aquatic product market	*Vibrio* spp.	Podovirus	dsDNA	43.5	OM799543	Broad	≤50 °C; pH 5–10	Aquaculture industry/food safety control	[129]
BPVP-3325	Seawater, wet sand, sea rocks, and suspended solids from Busan, South Korea	*V. parahaemolyticus*	Myovirus	dsDNA	222.6 *	NA	Broad	10–40 °C; pH 5–10	Oyster culture and food industry	[161]
vB_VnaS-L3	Marine aquaculture	*Vibrio natriegens* AbY-1805	Siphovirus	dsDNA	40.0	ON714422	Narrow	4–40 °C; pH 6–10	Abalone aquaculture	[127]
VA5	Aquaculture farms and sewage	*V. alginolyticus*	Siphovirus	dsDNA	35.9 *	NA	Broad	−20–70 °C; pH 2–10	Shrimp aquaculture	[131]
CAU_VPP01	Beach mud	*V. parahaemolyticus*	Siphovirus	dsDNA	79.8	OQ858564	Broad	≤60 °C; pH 4–10	Seafood industry	[149]
VB_VaC_TDDLMAVB_VaC_SRILMA	Water samples from Ilhavo channel, Aveiro, Portugal	*V. alginolyticus*	Myovirus	dsDNA	195.8 195.8	PP083315 PP083314	Narrow	-	Larviculture—live feed biocontrol	[58]
IKEM_vK IKEM_v5 IKEM_v14	Hatchery	*Vibrio* spp.	Siphovirus	dsDNA	NA NA NA	NA NA NA	Broad	≤60 °C; pH 5–11	Aquaculture industry	[179]
ɸTT1H ɸTT2H ɸA2223	Shrimp farm	*Desulfovibrio* spp. and *V. parahaemolyticus*	-	dsDNA	NA NA NA	NA NA NA	Broad	-	Shrimp aquaculture	[114]
P122 P125 P160	Cockles, oysters, water, soil sediments, shrimps, mussels, and green caviar	*V. alginolyticus*	Siphovirus	dsDNA	76.3 76.3 76.0	NA NA NA	Broad	25 °C	Aquaculture industry	[26]
vB_VpaS_1601 vB_VpaP_1701	Oysters	*V. parahaemolyticus*	Siphovirus Podovirus	dsDNA	78.5 44.0	OQ719603 ON872379	Broad	4–50 °C; pH 3–11	Food safety	[29]

* The genome size of the prophage was estimated using electrophoretic techniques such as PFGE and restriction digestion. NA in the Genome Size column indicates that the prophage’s molecular weight was not determined/reported. NA in the Accession Number column indicates that either the prophage genome was not sequenced, or if sequenced, it was not made publicly available.

## 4. Innovations in Marine Phage Therapy

Phage therapy’s resurgence in the post-antibiotic era presents new frontiers in the fight against antimicrobial resistance. Given the increasing prevalence of MDR pathogens, phage therapy offers huge potential in treating chronic infections, multidrug-resistant pathogens, and biofilm-associated infections [12,15]. The clinical relevance of phage therapy extends to veterinary care, food safety, and environmental management. However, despite its promise, phage therapy still faces various challenges, including regulatory hurdles, safety concerns, and the risk of bacterial resistance to phages [180]. Ongoing research and clinical trials are critical to addressing these problems and incorporating phage therapy into mainstream treatment options.

Recent innovations in phage therapy provide solutions to these challenges and enhance therapeutic outcomes. Synergistic therapy combines phages with antibiotics to reduce required dosages, delay resistance development, and enhance efficacy, particularly against hard-to-treat pathogens [181]. Bioengineered phages are genetically modified to expand host range, improve stability, and enhance lytic activity [182]. Phage-derived enzymes, especially endolysins, provide bactericidal activity targeting bacterial cell walls without live phages [183]. These breakthroughs pave the way for more efficient and safer therapeutic options, thereby promoting the integration of marine phage therapy into modern clinical and environmental practices.

### 4.1. Synergistic Effects with Antibiotics

Among the most promising developments in phage therapy is phage–antibiotic synergy (PAS), which refers to the combined use of phages and antibiotics to enhance bacterial killing. PAS leverages distinct yet complementary mechanisms of action to improve therapeutic efficacy, limit resistance development, and enhance clinical outcomes, particularly against chronic infections [181,184].

PAS typically involves antibiotics at sub-inhibitory concentrations enhancing phage replication dynamics, increasing adsorption rates, shortening latent periods, and enlarging burst sizes [185,186]. While studies predominantly used terrestrial models (e.g., β-lactams enlarging phage plaques in *E. coli* through filamentation), such principles remain instructive for marine systems, where similar mechanisms likely operate [187].

**Figure 7:**
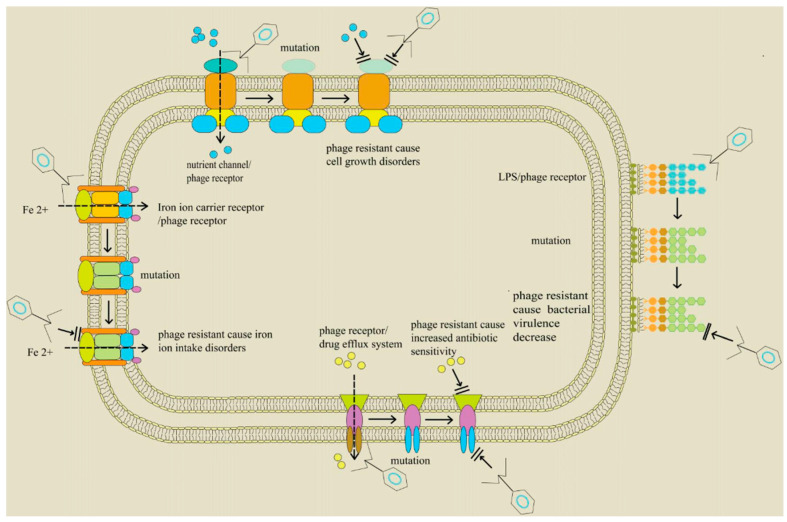
Evolutionary trade-offs of phage resistance in bacteria. Bacterial surface proteins, including nutrient channels, iron ion uptake receptors, lipopolysaccharides (LPS), and drug efflux pumps, serve as receptors for phage adsorption and entry. When bacteria acquire phage resistance through mutations in these proteins, they often experience physiological trade-offs. Mutations in nutrient channels and iron transporters can impair bacterial growth and iron acquisition, while alterations to LPS may reduce bacterial virulence. Changes in drug efflux pumps can block phage attachment but also increase bacterial susceptibility to antibiotics. These adaptations not only disrupt essential physiological functions but also sensitize bacteria to antibiotics, providing a mechanistic basis for phage–antibiotic synergy. Reprinted from [181], Copyright © 2021 by the authors and distributed under the terms of the Creative Commons Attribution License: https://creativecommons.org/licenses/by/4.0/.

Mechanistically, PAS in marine systems could potentially exploit evolutionary trade-offs similar to those observed in terrestrial pathogens. For example, phage resistance adaptations often involve mutations in bacterial surface proteins that serve as phage receptors, which can compromise essential functions such as nutrient uptake or antibiotic efflux, inadvertently increasing antibiotic susceptibility (Figure 7). Another possible mechanism is target diversification in which phages and antibiotics act on distinct bacterial targets (e.g., cell wall versus DNA replication), thereby reducing the risk of cross-resistance [181].

Though extensive PAS research is available for terrestrial pathogens, marine-specific studies remain limited and nascent. Manohar et al. [188] examined PAS using Citrophage MRM57, a Citrobacter-specific phage isolated from seawater in Ramanathapuram, Tamil Nadu, India. They reported that combining this phage at sublethal titers (10^3^–10^6^ PFU/mL) with various antibiotics, including β-lactams, aminoglycosides, carbapenems, and polymyxins, resulted in up to a 99.99% reduction in the nosocomial pathogen *Citrobacter amalonaticus*. Importantly, the phage remained stable and infectious in the presence of antibiotics tested, demonstrating its suitability for therapeutic application in marine-derived pathogens. Similarly, the TEMp-D1 phage, which infects *Photobacterium damselae* subsp. *damselae* (PDD), a pathogenic bacterium that affects both marine animals and humans, was shown to significantly enhance the inhibition of PDD biofilm formation and cell growth when applied in combination with sublethal concentrations of antibiotics such as oxytetracycline, florfenicol, sulfadiazine-trimethoprim, and enrofloxacin compared to either phage or antibiotic treatment alone [189]. Lopes et al. [190] also evaluated PAS using phage ELY-1 isolated from aquaculture facility and showed that its combination with ciprofloxacin at MIC effectively reduced bacterial density and the emergence of resistant mutants compared to single treatments. These studies highlight the need to expand PAS research in marine environments in order to harness the full therapeutic potential of marine phages for aquaculture health management and biomedical applications.

### 4.2. Bioengineered Marine Phages

Natural phages hold promise for biocontrol and therapy but face limitations such as a narrow host range, moderate lytic activity, and the potential for bacterial resistance [191,192]. Advances in synthetic biology and molecular engineering have enabled precise modification of phage genomes to address these challenges [33]. Host range engineering by modifying RBPs is a leading strategy to overcome resistance linked to bacterial surface variability. While this approach has shown significant progress in terrestrial phage models [193,194], its broader application to marine phages could be promising but is currently limited by the need for a deeper understanding of marine phage–host receptor interactions.

**Figure 8:**
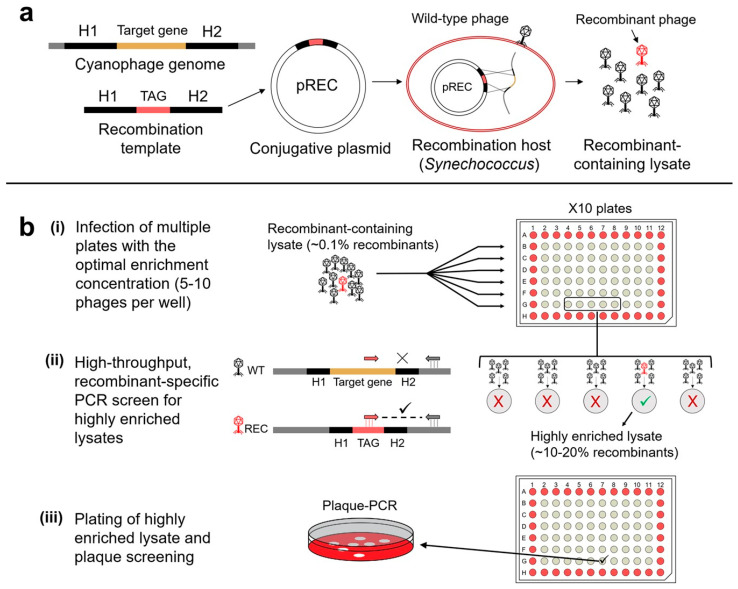
A schematic overview of the REEP (Recombination, Enrichment, and Engineering of Phages) genetic engineering system for marine cyanophages. (**a**) The construction of recombinant phages is carried out as follows: A recombination template is designed with two homologous regions (H1 and H2, each 200–300 base pairs long) flanking the target gene in the cyanophage genome. A short TAG sequence is also included, which will be integrated in place of the target gene. This template is cloned into the pREC plasmid and introduced into a *Synechococcus* host cell, creating a recombination host. The infection of this host with wild-type cyanophages allows homologous recombination to take place, generating a mixed phage lysate containing both wild-type (depicted in black) and recombinant (depicted in red) phages. (**b**) Enrichment and screening for recombinant phages is carried out as follows: (i) The recombinant-containing lysate is distributed into multiple 96-well plates, with each well being infected using an optimal enrichment concentration of 5–10 phages per well. (ii) After lysis, a high-throughput, recombinant-specific PCR assay is performed on each well to identify lysates that are highly enriched for recombinant phages. Highly enriched lysates are defined as those in which recombinant phages constitute more than 10% of the total phage population, representing a greater than 100-fold increase compared to the initial frequency (0.1%). (iii) Lysates that test positive in the PCR screen are then plated for plaque assays. Individual plaques are screened by PCR to confirm the presence and isolation of the recombinant phage. Reprinted from [102], Copyright © 2021 by the author(s) and licensed under a Creative Commons Attribution 4.0 International License (http://creativecommons.org/licenses/by/4.0/).

Therapeutic payload delivery represents another promising approach for engineering phages. Incorporating genes that encode antimicrobial effectors, such as biofilm-degrading enzymes, quorum-quenching proteins, or peptidoglycan hydrolases, enables engineered phages to disrupt protective biofilms and enhance bacterial killing [195,196,197]. Engineered phages can also deliver intracellular payloads, including antimicrobial peptides, toxins, or gene modulators, that disrupt essential bacterial processes, such as genome replication and protein synthesis [33]. Shitrit et al. [102] also developed a robust phage genome engineering system (REEP) for marine cyanophages infecting *Synechococcus* and *Prochlorococcus*, demonstrating the feasibility of targeted genome editing in marine phages and uncovering unique infection strategies, including transient integration without stable lysogeny (Figure 8). The conversion of temperate phages into strictly lytic forms, via the deletion of lysogeny-associated genes, will improve the therapeutic profile of candidate phages by reducing the risk of HGT and enhancing killing efficiency. Moreover, modifications to remove virulence genes or produce lysis-deficient phages offer increased safety by minimizing endotoxin release and proinflammatory responses during therapy [182].

### 4.3. Phage-Derived Enzymes

Phage-derived enzymes, such as endolysins and depolymerases, have also emerged as promising alternatives for combating microbial infections. These enzymes, either naturally occurring or bioengineered, exert potent lytic activity and can function independently of whole phage particles. Marine phage-derived enzymes are gaining attention as a result of their evolutionary adaptation to a broad range of Gram-negative hosts, particularly within the genus *Vibrio*, which are frequent causative agents of infections in aquaculture systems [183,198,199].

**Figure 9:**
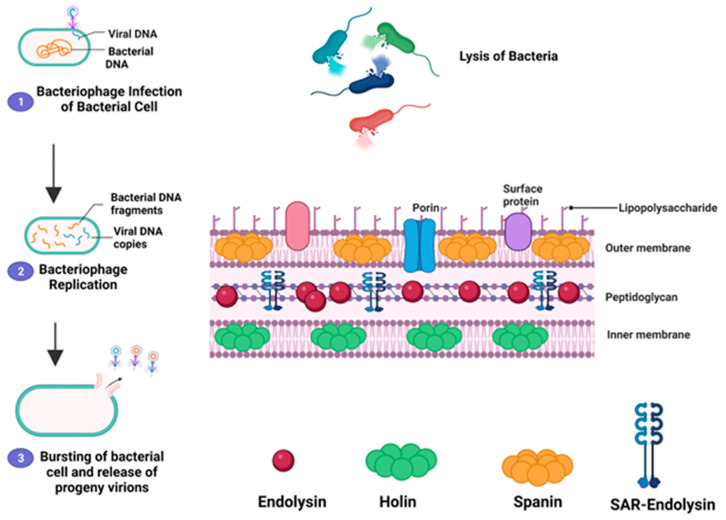
The mechanism of action of phage-encoded endolysins in Gram-negative bacteria cell lysis. The schematic illustrates the mechanism of action during the lytic cycle of bacteriophage and phage-derived endolysins in Gram-negative bacteria: (1) The bacteriophage recognizes and attaches to specific receptors on the bacterial surface via its tail fiber proteins. (2) Following attachment, the phage injects its genetic material into the host cell, where the phage genome is replicated and new progeny phages are assembled; holins accumulate in the inner membrane and form channels, allowing endolysins to access and degrade the peptidoglycan layer. In some phages, alternative lysis proteins such as pinholins and signal-anchor-release (SAR) endolysins are employed, with pinholins triggering the release of membrane-tethered SAR endolysins. Spanin proteins are also produced and assemble across the membranes. (3) In the final stage, endolysins degrade the peptidoglycan, spanins disrupt the outer membrane, and the bacterial cell undergoes lysis, releasing new phage progeny. Reprinted from [203], Copyright © 2023 Khan, Chen, Zhang, and Liu, which is distributed under the terms of the Creative Commons Attribution License: https://creativecommons.org/licenses/by/4.0/.

The release of phage progeny at the end of the lytic cycle is generally orchestrated by holins and endolysins. Holins accumulate in the bacterial cytoplasmic membrane during the late phase of infection, forming pores that allow endolysins to access and cleave specific bonds within the peptidoglycan layer, ultimately resulting in cell lysis (Figure 9). In Gram-negative hosts, however, complete lysis additionally requires the action of spanins to disrupt the outer membrane. In addition to the classical holin–endolysin pathway, some Gram-negative phages utilize alternative lysis mechanisms, such as the pinholin–signal-anchor-release (SAR) endolysin system, in which pinholins form small membrane lesions that activate and release membrane-tethered SAR endolysins. [198]. Although the holin–lysin lysis system is well established in bacteriophages, recent genomic studies of marine phages such as *V. parahaemolyticus* phage VPp1 and phage qdvp001 have revealed the absence of identifiable holin genes in their genomes [200,201]. This observation parallels findings in bacteriophage Mu, where lysis occurs via an alternative, holin-independent pathway. Instead, phage Mu utilizes a SAR endolysin, whose release and activation are mediated by a membrane-tethered protein called a “releasin” (gp25) [202].

LysVPp1, an endolysin encoded by the marine phage VPp1, displays transglycosylase activity and a broader lytic spectrum than its parental phage by effectively lysing nine of twelve *Vibrio* strains tested. Its activity is improved in the presence of membrane permeabilizers like EDTA, which help the enzyme reach the peptidoglycan layer, one of the barriers in Gram-negative bacteria [200]. Similarly, phage R16F, infecting the same pathogen, encodes an endolysin with high sequence identity to Lysqdvp001, a potent enzyme known for its lytic activity against numerous *Vibrio* strains [204]. Lysqdvp001 features a Cysteine, Histidine-dependent Amidohydrolase/Peptidase (CHAP) catalytic and a peptidoglycan-binding domain, a combination that enhances both substrate specificity and catalytic efficiency. It lysed all 11 tested *V. parahaemolyticus* strains, far surpassing its parent phage’s narrow host range [205].

In addition, LysVPMS1 obtained from a phage isolated during an outbreak of acute hepatopancreatic necrosis disease (AHPND) exhibited lytic activity against both AHPND and non-AHPND *V. parahaemolyticus* isolates, as well as other marine pathogens including *V. alginolyticus*, *V. harveyi*, and *V. campbellii* [206]. Likewise, the endolysin from phage F23s1 efficiently reduced MDR *V. parahaemolyticus* counts within 60 min and also lysed *Salmonella* [207].

Recent work has revealed that marine phage endolysins can be successfully expressed in *Pichia pastoris* to synthesize enzymes with good stability and antimicrobial properties for aquaculture use. Vplys60, sourced from *V. parahaemolyticus* phage qdv001, exhibited strong lytic activity across a broad range of pH levels (6–10), temperatures (37–75 °C), and salinity (100–600 mM NaCl), with calcium ions substantially enhancing its effectiveness. It has strong anti-biofilm capacity, achieving 91.6% inhibition and concurrently reducing bacterial load in *Artemia franciscana* [208]. Similarly, LysVPB, also expressed in *P. pastoris*, was phylogenetically distinct and exhibited peak lytic activity at pH 9.0 and 30 °C, with calcium boosting its efficacy to 126.8% [209]. Both enzymes have strong specificity for *V. parahaemolyticus* and possess modular structures that allow for domain fusion and the creation of chimeric variants, making them promising tools for targeted antimicrobial applications in aquaculture.

In addition to endolysins, marine phages encode capsular depolymerases, though their therapeutic use remains underexplored. These enzymes break down bacterial capsules and biofilm matrices, thus improving access for immune components and antimicrobial agents. Although reports from marine phages are still limited, annotated genomes usually reveal tail fiber proteins and polysaccharide-degrading domains, pointing to a largely untapped resource for anti-biofilm strategies [210].

Only a few recombinant endolysins (e.g., SAL200, CF-301, and P128) have reached clinical or veterinary trials, where they have shown favorable safety profiles, immunotolerance, and efficacy against MDR pathogens [198,211]. On the other hand, artilysins and innolysins are extending the therapeutic applicability of these enzymes to Gram-negative pathogens by overcoming the outer membrane barrier without the need for chemical adjuvants [198].

There is growing evidence of the in vivo efficacy of endolysins against bacterial infections, although several challenges limit their translational potential. Their application against Gram-negative pathogens is challenged by the impermeable outer membrane, thus necessitating protein engineering or the use of membrane-permeabilizing agents [212,213]. Clinical translation remains difficult due to concerns like immunogenicity, poor tissue penetration, and rapid clearance from the body [214]. Current regulatory frameworks and drug approval pathways are not fully equipped to accommodate phage-based therapies, which delay clinical adoption [215]. Nevertheless, continued innovation in formulation and delivery strategies holds promise for overcoming these barriers and realizing its full therapeutic potential [216].

## 5. Challenges in Phage Therapy

Despite renewed interest and accumulating evidence of efficacy from animal studies and isolated human cases, phage therapy continues to encounter a number of barriers that hinder its widespread clinical application. Although phages are regarded as promising alternatives to conventional antibiotics, particularly in the context of escalating antimicrobial resistance, the transition from experimental models to routine clinical use remains a considerable challenge. One of the most pressing concerns is the rapid emergence of phage-resistant bacterial strains, a phenomenon that undermines therapeutic efficacy and mirrors the resistance crisis seen with antibiotics [217,218]. Moreover, the complex pharmacokinetic profile of phages within the human body, along with possible immunological responses, complicates their safe and effective deployment [219].

The remarkable specificity of phages, while beneficial for targeted therapy, also creates practical difficulties. Treating polymicrobial infections or those caused by heterogeneous bacterial populations often necessitates the formulation of large phage cocktails [220]. Moreover, issues such as the rapid clearance of phages by the host immune system and the unintended release of bacterial endotoxins upon lysis present further biological and safety challenges. Regulatory hurdles, the absence of standardized protocols for dosing and delivery, and the requirement for rigorous clinical validation remain major barriers to mainstream implementation [221]. Overcoming these challenges is essential to realizing the full therapeutic potential of phage therapy, both as a stand-alone intervention and as an adjunct to antibiotics [222].

### 5.1. Development of Phage-Resistant Bacteria

Phage therapy faces an inherent challenge as bacteria can rapidly evolve mechanisms to withstand phage infection. Research has demonstrated that bacterial populations frequently generate phage-resistant mutants, often with substantial genetic diversity [223]. Bacterial defense strategies are multifaceted, ranging from surface receptor alterations and superinfection exclusion systems to restriction–modification and abortive infection mechanisms [224]. Among the most sophisticated are CRISPR-Cas systems, which function as adaptive immune responses that enable bacteria to recognize and neutralize invading genetic elements such as phage genomes and plasmids [225]. In *Vibrio* species, CRISPR-Cas systems are predominantly found on mobile genetic elements and exhibit diverse types and architectures [226].

*V. alginolyticus* can develop resistance to lytic phages through metabolic and membrane adaptations, including the downregulation of genes encoding outer membrane proteins (e.g., OmpF, LamB, and BtuB). This impairs phage adsorption but often comes with physiological trade-offs [153]. Similarly, in *V. anguillarum*, phage resistance is associated with diverse phenotypic changes and can result in reduced virulence, though some resistant strains may maintain pathogenicity [151,227]. Additionally, phage–host interactions may influence biofilm formation, with some phages promoting biofilm development as a bacterial defense strategy [228].

Leveraging fitness costs therapeutically can help steer bacterial evolution toward less pathogenic or more treatable phenotypes [229,230]. Moreover, a notable association has been observed between the degree of initial antibiotic resistance and the propensity for developing stable phage resistance, particularly in MDR strains [223]. These findings emphasize the importance of co-evolutionary dynamics in phage therapy, where constant adaptation on both sides requires ongoing innovation in therapeutic strategies and phage cocktail design.

### 5.2. Pharmacokinetic and Pharmacodynamic Complexities

Unlike conventional antibiotics, phages are self-amplifying agents, meaning their concentrations within the host are shaped not only by the administered dose but also by their capacity to multiply at the site of infection, provided susceptible bacteria are present [231,232]. This introduces unique variables, such as multiplicity of infection, the distinction between passive versus active therapy, and the influence of host immune responses, which further complicate traditional PK/PD modeling [232]. Phage pharmacokinetics cover sophisticated processes of absorption, distribution, metabolism, and elimination [233]. Achieving sufficiently high “killing titers” is a critical determinant of successful phage therapy; however, depending solely on in situ phage replication may not ensure bacterial eradication, particularly in situations where phage particles are inactivated or fail to adequately reach the infection site [234].

Kim et al. [146] found that only high concentrations of certain phages provided significant protection against *V. coralliilyticus* infection in Pacific oyster larvae, with lower doses showing little effect. Similarly, Lal et al. [22] demonstrated that the lytic phage VhKM4 displayed strong activity against *V. harveyi* at high MOI, but only delayed or partial effects at lower MOIs. Rørbo et al. [143] showed that the application of the broad-host-range phage KVP40 reduced or delayed mortality in cod and turbot larvae challenged with various *V. anguillarum* strains, yet efficacy varied by strain and the effect was often temporary, likely due to ecological interactions with the background microbiota.

Mathematical modeling now plays an increasingly important role in optimizing phage dosing strategies as it allows for the integration of preclinical data and the simulation of complex interactions among phages, bacteria, and host immune responses [235]. Nevertheless, the absence of standardized methodologies and gaps in understanding in vivo pharmacokinetics and pharmacodynamics continue to limit reproducibility and impede the development of rational, effective phage therapy protocols [236], stressing the pressing need for further research and a harmonized clinical framework.

### 5.3. Host Immune Responses to Phage Therapy

Phages are inherently immunogenic. Upon entering the host, they interact with both innate and adaptive immune components [237]. In fish, for example, phage abundance in the mucosal layer can confer protection against bacterial pathogens, yet these same immune barriers, like phagocytic cells and pattern recognition receptors, may obstruct phage access to their targets and contribute to rapid clearance from the host system [238]. Kalatzis et al. [24] have highlighted that in aquaculture species, the activation of the adaptive immune system can result in the rapid removal of phages from circulation, potentially preventing them from reaching and lysing target bacteria at infection sites.

In addition, phages may exhibit immunomodulatory effects in aquatic animals, including the suppression of proinflammatory cytokines in certain contexts, but they may also stimulate robust immune responses depending on the formulation and delivery [239,240,241]. The risk of immune clearance must also be balanced with concerns that rapid bacterial lysis can release endotoxins, potentially exacerbating inflammation [237,238]. While immune evasion strategies such as phage encapsulation or chemical modification are being explored [136,242], optimizing phage therapy for marine and aquaculture applications requires careful consideration of host immune responses to ensure effective and sustained antibacterial activity.

### 5.4. Phage Specificity

A primary drawback of phage therapy is the limited host range characteristic of most phages, which often restricts their activity to specific genera, species, or even particular strains. Although this precision helps preserve the natural microbiota, it complicates treatment scenarios where infections may be caused by genetically diverse or multiple bacterial pathogens [220,243]. In agricultural settings such as food animal production, traditional phage therapy approaches that rely on cocktails of multiple phages become increasingly impractical. As livestock often harbor a broad zoonotic pathogen, effective control would demand the formulation of cocktails containing dozens of different phages. This requirement introduces pronounced logistical challenges and increases the overall cost of implementation, especially at commercial scales [220].

The restricted host range of phages also means that preparations must be carefully matched to the infecting pathogen(s), which calls for either rapid diagnostics or ready access to broad and diverse phage libraries to ensure timely and appropriate treatment [168]. In addition, the high specificity of individual phages can impede their utility in empirical therapy, where the causative agent is not immediately identified [244]. Notably, resistance to single phages tends to arise swiftly when only one bacterial receptor is involved, but the use of phage mixtures that recognize a variety of receptors proved far more successful in delaying the emergence of resistant strains [245]. These cases highlight both the remarkable specificity of phages and the need for comprehensive host range characterization and strategic cocktail design to enable the practical and effective use of phage therapy in both clinical and agricultural settings.

### 5.5. Safety Concerns

Even successful phage infection and lysis of its host have consequences, particularly when treating infections caused by Gram-negative bacteria, such as *Vibrio* [220]. Phage-mediated lysis disrupts the bacterial cell wall, resulting in the release of endotoxins, especially the lipid A component of lipopolysaccharide (LPS), into the extracellular environment [12]. This rapid release of endotoxin can provoke strong inflammatory reactions in the host and, under certain circumstances, like higher bacterial load, may lead to endotoxemia or even septic shock [246].

Comparative studies have demonstrated that phage-induced lysis releases lower quantities of endotoxin than conventional β-lactam antibiotics, suggesting a relatively safer profile in this regard [247]. Nevertheless, the potential for systemic adverse effects persists. Findings from murine studies indicate that treatment with oral phage cocktails can enhance intestinal permeability and elevate plasma endotoxin concentrations, thus increasing the risk of developing endotoxemia [248].

Progress in phage purification techniques now allows for the production of formulations with the endotoxin content reduced to levels that comply with established safety standards for intravenous administration [249]. An alternative and increasingly promising strategy entails the use of genetically engineered or naturally occurring nonlytic phages. Filamentous phages such as M13 and Pf3, as well as engineered lysis-deficient variants, can eliminate bacterial cells without causing cell lysis, resulting in a substantially lower amount of endotoxin released [250,251]. Comparative analyses demonstrate that infections treated with nonlytic phages yield significantly lower endotoxin levels than those treated with traditional lytic phages, both in vitro and in animal models. In vivo, these strategies have been linked with reduced inflammatory responses and improved survival outcomes, especially under high bacterial load conditions [220]. Lastly, endolysin gene knockouts have been engineered in phages such as T4 and P954. Although these endolysin-deficient phages are unable to release progeny, they still maintain bactericidal activity by utilizing holins to form pores in the bacterial inner membrane [252].

These innovations in phage purification and engineering represent critical steps toward increasing the therapeutic index and clinical safety of phage-based interventions for multidrug-resistant Gram-negative infections. Continued progress in this area is expected to further reduce the risk of phage-induced endotoxemia and broaden the clinical applicability of phage therapy.

### 5.6. Delivery Systems and Dosage Optimization

The stability of phages during storage and delivery remains a critical bottleneck to their widespread application in food safety, aquaculture, and therapeutic interventions [253]. Although marine phages often demonstrate enhanced tolerance to environmental fluctuations compared to terrestrial phages, they remain susceptible to various physicochemical stresses encountered during processing and clinical deployment. Phages targeting *V. parahaemolyticus* retain infectivity at moderate pH (5–9) but experience rapid titer reduction below pH 4 or after exposure to simulated gastric fluids (pH 1.2), where no viable particles are detectable after 60 min [130,254]. Likewise, most marine phages are inactivated at temperatures above 60 °C [149,179], presenting a challenge for both storage in warm climates and their integration into processed therapeutic formulations.

Phages are also sensitive to environmental factors such as ultraviolet (UV) irradiation and desiccation, which are usually encountered during storage, transportation, and field-based therapeutic administration [253]. *Vibrio* phages can lose viability within minutes of UV-C exposure, which limits their effectiveness in open-water or pre-harvest aquaculture applications unless appropriate protective measures are in place [130]. In light of these, studies have increasingly focused on advanced formulation and encapsulation strategies to improve phage stability throughout storage and delivery. Encapsulating phages in protective matrices, such as alginate, chitosan, or liposomes, can shield them from hostile conditions, including acidic pH, UV irradiation, and desiccation [255,256,257]. These systems not only increase phage survival during long-term storage and transit but also facilitate controlled or targeted release at infection sites, thus improving therapeutic outcomes. Desiccation processes like spray-drying and freeze-drying, though essential for generating stable phage powders for long-term storage or for oral and injectable preparations, can result in significant loss of infective units unless protective excipients such as trehalose or sucrose are incorporated [136,253]. Customized formulation strategies that not only stabilize phages during storage and transit but also ensure their safe and effective delivery to infection sites should continue to be explored to advance their clinical translation and broaden their therapeutic applications.

### 5.7. Regulatory and Standardization Challenges

Despite the resurgence of interest in phage therapy as a viable solution to the global antibiotic resistance crisis, substantial regulatory and standardization obstacles remain, particularly within Western medical frameworks. At present, no commercial phage products for *Vibrio* control exist yet, reflecting regulatory gaps [129]. The foundation of modern drug regulation is based on the evaluation of industrial-scale, chemically defined pharmaceuticals, which fundamentally differs from the biological complexity, variability, and personalization that characterize phage therapy [258].

One major setback is the lack of appropriate and universally accepted animal models for preclinical testing. Phage pharmacokinetics, host interactions, and therapeutic outcomes can vary significantly from conventional drugs, necessitating dedicated models to assess efficacy and safety in vivo. Also, regulatory authorities require rigorous, standardized clinical trials to validate safety and efficacy, a process complicated by the bespoke nature of phage preparations and the need to rapidly tailor cocktails to individual infections or outbreaks [221,259].

Current “one-size-fits-all” pharmaceutical regulations are not structured to address the adaptive and typically patient-specific requirements of phage therapy. Each batch of phages may be unique, and production must take into consideration viral evolution, genetic stability, and risk of contamination, making it difficult to develop clear quality control, manufacturing standards, and dosing protocols [260].

Nevertheless, efforts to establish dedicated regulations are underway, with some countries revisiting approaches from nations where phage therapy has been continuously practiced [261]. Addressing these regulatory and standardization barriers entails coordinated efforts among clinicians, researchers, regulatory bodies, and industry stakeholders. Transparency in clinical data reporting, consensus on quality control measures, and the development of streamlined, flexible regulatory pathways are prerequisites for the safe and effective adoption of phage therapy [262]. Without these regulatory and procedural changes, the routine clinical use of phage-based interventions is likely to remain limited, even as their capacity to revolutionize the next generation of antimicrobial treatment becomes increasingly evident.

## 6. Conclusions

The escalating challenge of antimicrobial resistance among bacterial pathogens has intensified the search for effective alternatives to conventional antibiotics, with marine environments emerging as a rich source of novel solutions. Marine bacteriophages stand out as some of the most promising and safe options for combating infections, owing to their remarkable specificity, potent lytic activity, and adaptability to extreme aquatic conditions. This review has highlighted the vast diversity of marine phages, their complex host interactions, and their diverse mechanisms for eliminating bacterial pathogens.

The practical value of marine phage therapy is further supported by its demonstrated effectiveness against multidrug-resistant bacteria, ability to disrupt biofilms, attenuation of bacterial virulence, and enhancement of host survival, particularly within aquaculture systems. Recent advances include the synergistic use of phages with antibiotics, which offers heightened efficacy against resistant strains, and the bioengineering of phages to broaden host range and boost lytic potential. In addition, phage-derived enzymes present an expanding frontier by delivering highly specific and efficient antibacterial action.

These innovations collectively address some of the longstanding limitations of natural phages, broadening their therapeutic scope and enabling the development of personalized approaches to bacterial infection management. As research continues to unlock the potential of marine phages, their integration into mainstream therapeutic pipelines offers a promising pathway to control persistent and emerging bacterial threats across clinical, aquaculture, and environmental settings.

## 7. Future Perspectives

The following are the future directions that should be prioritized to advance the development and clinical translation of marine phage therapy:

1. Expanded exploration and the genomic characterization of marine environments should be conducted to harness the largely untapped diversity of marine phages. While high-throughput metagenomics and advanced bioinformatics will continue to uncover novel phages with unique infection mechanisms and broad-spectrum activity, integrating multi-omics approaches will be essential for a more comprehensive understanding of phage–host interactions. Proteomic and metabolomic profiling of phage-infected cells can reveal dynamic changes in host cellular machinery, identify functional viral proteins (including those subject to post-translational modifications), and clarify the biochemical pathways impacted during infection. These will accelerate the translation of marine phages into therapeutic pipelines targeting multidrug-resistant pathogens by not only identifying candidates but also elucidating their mechanisms of action at the systems level.
2. The development of adaptive, rationally designed phage cocktails should be emphasized to delay or prevent the emergence of phage-resistant bacteria. Incorporating insights from co-evolutionary dynamics and host range profiling will enable the formulation of phage mixtures that leverage evolutionary trade-offs, attenuate bacterial virulence, and restore antibiotic susceptibility.
3. Comprehensive pharmacokinetic and pharmacodynamic studies, supported by mathematical modeling and standardized protocols, are essential for optimizing marine phage dosing protocols, ensuring reproducibility, and accurately predicting therapeutic outcomes.
4. Efforts to advance encapsulation and delivery systems, particularly those utilizing liposomal or polymer-based carriers, should be expanded to improve marine phage stability, increase resistance to immune clearance, and allow precise, site-specific delivery in therapeutic applications.
5. The establishment and curation of expansive marine phage libraries, along with rapid molecular diagnostics and high-throughput host range screening, will facilitate the timely and precise customization of phage therapies for diverse and emerging bacterial pathogens. Continued focus on robust host range prediction and systematic production standardization is necessary for effective and reliable therapeutic deployment.
6. Synthetic biology and genetic engineering approaches should be harnessed to expand phage host range, lower immunogenicity, and develop nonlytic or lysis-deficient marine phage variants that attenuate endotoxin release and reduce the risk of inflammatory complications, especially in the treatment of Gram-negative infections.
7. International collaboration is needed to create dedicated regulatory frameworks and harmonized quality standards for marine phage therapeutics. This includes developing adaptive manufacturing protocols, robust characterization procedures, and streamlined clinical evaluation pathways to facilitate safe and effective marine phage therapy implementation worldwide.
8. Multidisciplinary strategies that leverage advances in synthetic biology, systems microbiology, and environmental virology can expedite the translation of marine phage therapy from laboratory research to mainstream application. Employing these approaches will improve host range prediction, resistance management, scalable manufacturing, and rigorous in vivo safety and efficacy assessments.
9. Greater emphasis on the mechanistic details of interactions, such as phage-mediated biofilm disruption, the modulation of bacterial resistance pathways, and effects on host immunity, when combining marine phages with antibiotics, probiotics, and phage-derived enzymes, may help refine and optimize combination strategies and limit the emergence of resistance.

## Data Availability

No data are associated with this study.
